# Profile of *Trypanosoma cruzi* Infection in a Tropical Medicine Reference Center, Northern Italy

**DOI:** 10.1371/journal.pntd.0003361

**Published:** 2014-12-11

**Authors:** Federico Gobbi, Andrea Angheben, Mariella Anselmi, Chiara Postiglione, Ernestina Repetto, Dora Buonfrate, Stefania Marocco, Stefano Tais, Andrea Chiampan, Paride Mainardi, Zeno Bisoffi

**Affiliations:** 1 Centre for Tropical Diseases (CTD), Sacro Cuore Hospital, Negrar, Verona, Italy; 2 Centre for Community Epidemiology and Tropical Medicine (CECOMET), Esmeraldas, Ecuador; 3 Prevention Department, ULSS 20, Verona, Italy; 4 Operational Center Bruxelles, Médecins Sans Frontières, Bruxelles, Belgium; 5 Cardiology Unit, Sacro Cuore Hospital, Negrar, Verona, Italy; 6 Radiology Unit, Sacro Cuore Hospital, Negrar, Verona, Italy; Universidad de Buenos Aires, Argentina

## Abstract

**Background:**

Chagas disease (CD) is endemic in Central and South America, Mexico and even in some areas of the United States. However, cases have been increasingly recorded also in non-endemic countries. The estimated number of infected people in Europe is in a wide range of 14000 to 181000 subjects, mostly resident in Spain, Italy and the United Kingdom.

**Methodology/Principal Findings:**

Retrospective, observational study describing the characteristics of patients with CD who attended the Centre for Tropical Diseases (Negrar, Verona, Italy) between 2005 and 2013. All the patients affected by CD underwent chest X-ray, ECG, echocardiography, barium X-ray of the oesophagus and colonic enema. They were classified in the indeterminate, cardiac, digestive or mixed category according to the results of the screening tests. Treatment with benznidazole (or nifurtimox in case of intolerance to the first line therapy) was offered to all patients, excluding the ones with advanced cardiomiopathy, pregnant and lactating women. Patients included were 332 (73.9% women). We classified 68.1% of patients as having Indeterminate Chagas, 11.1% Cardiac Chagas, 18.7% as Digestive Chagas and 2.1% as Mixed Form. Three hundred and twenty-one patients (96.7%) were treated with benznidazole, and most of them (83.2%) completed the treatment. At least one adverse effect was reported by 27.7% of patients, but they were mostly mild. Only a couple of patients received nifurtimox as second line treatment.

**Conclusions/Significance:**

Our case series represents the largest cohort of *T. cruzi* infected patients diagnosed and treated in Italy. An improvement of the access to diagnosis and cure is still needed, considering that about 9200 infected people are estimated to live in Italy. In general, there is an urgent need of common guidelines to better classify and manage patients with CD in non-endemic countries.

## Author Summary

Chagas disease is endemic in Central and South America, Mexico and part of the United States. However, migration flows permitted a diffusion outside those borders, so nowadays there are infected people living in Europe, mostly in Spain, Italy and the United Kingdom. This manuscript describes the patients with Chagas disease attended at a referral center in Italy between 2005 and 2013. They were classified into different categories according to the presence of possible cardiac and/or gastrointestinal involvement: one third of patients presented signs of organ damage. Ninety-seven percent of the infected patients were treated with benznidazole, 16% had to stop the treatment due to adverse events.

## Introduction

Chagas disease (CD) is a protozoan zoonosis caused by *Trypanosoma cruzi* (*T.cruzi*), with a widespread distribution from the South of the United States to Mexico, Central and South America [1]. Current data indicate that between 7 and 8 million people are infected in this area [2]. Other authors suppose that in North America (Canada, USA, Mexico) there might be many more infected subjects than it was previously estimated (1 to 6 million people) [3], therefore global estimations should be revised. In endemic countries, *T. cruzi* infection is usually transmitted through contact with faeces of blood-sucking triatomines, rarely after oral ingestion of food contaminated by triatomines faeces. Other non-vectorial routes of transmission are transplacentary, blood transfusion and organ/tissue transplantation [1].

The acute stage of the infection is frequently asymptomatic (95% of cases); when symptomatic, acute CD can cause myocarditis and encephalomyelitis with reported 5–10% mortality [1]. If not treated, the acute phase is followed by a chronic stage, corresponding to an indeterminate form, lasting long-life in around 60–70% of patients. During this phase patients are clinically silent: however, after 10 to 30 years, around 30–40% of infected people will develop symptomatic chronic CD, which is mainly characterized by cardiac and gastrointestinal disorders [1].

As a consequence of migration flows, the disease has been increasingly recorded also in non-endemic countries and is becoming a global health problem [4]. The estimated number of infected people in Europe is in a wide range of 14,000 to 181,000 subjects [5]. Italy has a large number of residents of Latin American origin, second in Europe only to Spain [6]. Strasen *et al* estimate that in Italy there are about 9,200 cases (range 1,400–17,000) [5].

The majority of Latin American migrants reached Italy in the past ten years, with a growing trend. Migrants from different countries tend to have a patchy distribution in Italian Regions, with a major concentration in Northern Italy and in Rome. For instance most Bolivians live in Bergamo Province, Lombardy, Ecuadorians in Liguria Region, and Peruvians in big cities such as Milan, Florence and Rome [6].

The Centre for Tropical Diseases (CTD), Hospital Sacro Cuore (Negrar, Verona) contributed to describe this new epidemiological scenario and became a reference center in the management of CD.

Aim of this paper is to describe the characteristics of patients with CD attended at our center.

## Methods

### Study design

This is a retrospective, observational study, including all patients with CD attended at CTD between 2005 and 2013. Most of these patients were identified through a specific screening programme offered by our center to Latin American communities resident in Northern Italy; moreover, other patients were referred from other centers to the CTD on the basis of clinical suspicion. Up to 2014, there were no other screening programmes in Italy focused on CD.

Information collected included clinical and epidemiological characteristics such as: age, gender, country of origin, history of living in rural environments and mud houses, time since arrival to our country, history of blood transfusion in endemic countries, co-morbidities causing immunosuppression, cardiac and gastrointestinal assessment, completion of treatment. Data were anonymized and entered in an Epi-Info database.

Chagas Disease diagnosis was based on two concordant positive serological tests, using different antigens, a recombinant antigen-based ELISA (BioELISA Chagas, Biokit, Lliça d′Almunt, Spain) plus either a *T.cruzi* lysate antigen-based ELISA (BioELISA Chagas III, BiosChile, Santiago, Chile) or an immunochromatographic assay (Chagas Quick Test, Cypress Diagnostics, Belgium). As recommended by the World Health Organization [7], in case of discordant result, a third assay (an immunoblot, TESA-cruzi) [8] was performed. Patients with CD were examined following an internal protocol that included haematology and biochemistry. Cardiac involvement was assessed with chest X-ray, electrocardiogram (ECG), echocardiography [9]. Chest X-ray was considered pathological in case of cardiac enlargement, defined as cardiothoracic ratio greater than 0,5. The ECG was evaluated by a cardiologist and considered pathological in case of bradycardia <50 bpm, atrio-ventricular block, bundle branch block or hemiblock, low voltages, tachyarrhythmia of any origin, Q-waves suggestive of necrosis and T-waves changes suggesting ischemia. The echocardiography was considered pathological in case of systolic dysfunction, defined as a left ventricular ejection fraction (LVEF) below 45%, segmental abnormalities of myocardial contraction, presence of aneurismas (typically apical), dilated cardiomyopathy [9].

The modified Brazilian classification was followed for cardiac CD classification [10].

Digestive involvement was assessed with barium X-ray of the oesophagus (oesophagogram) [11] and colonic enema [12].

Oesophagogram was considered pathological following Rezende's classification [11].

Barium enema was defined pathological in case of megacolon (descending colon diameter>6,5 cm or ascending colon diameter>8 cm, or caecum diameter>12 cm) [13] or dolichocolon (length from the anus to the transition to the descending colon>70 cm) [13].

*T. cruzi* infected patients without cardiac or digestive involvement were classified as Chagas indeterminate form; patients with cardiac or digestive involvement were classified respectively as Chagas cardiac form or Chagas digestive form, while patients with both cardiac and digestive form were classified as Chagas mixed form.

Specific treatment with benznidazole at 5 mg/kg/day divided into three daily doses for 60 days was offered to all infected patients, after careful explanation of benefits and risks of the therapy. Although current guidelines [14] recommend to offer the treatment to patients under 50 years of age, we extended the age limit up to 60 year-old patients, in the absence of severe comorbidities. Patients with advanced cardiomyopathy, pregnant (pregnancy test was performed in all women of child-bearing age before treatment) and lactating women were excluded. In patients weighing more than 60 kg, a fixed daily dose of 300 mg was given for a total number of days equal to the patient's weight in kilograms, resulting in a total dose that is equivalent to 5 mg/kg per day for sixty days [15]. In case of important early side effects due to benznidazole, nifurtimox (10 mg/kg/day for 60 days) was prescribed. Patients were considered adequately treated when the drug was taken for at least one month [16]. In case of mild adverse effects, continuation of treatment was encouraged, while benznidazole was stopped in case of severe adverse effects: for instance, severe allergic skin reactions, peripheral neuropathy, leucopoenia (WBC <2500 cells/µL).

Patients underwent a blood test (cell blood count and general biochemistry) at the beginning of the treatment and after 21 days. At day 30 they came for follow up visit to our outpatient clinic: if no significant laboratory or clinical adverse events were recorded, the remaining tablets were given to the patient for the completion of treatment. During the second month of treatment, follow-up was done through phone call or visit according to physician's judgement. We assessed the adherence to the treatment on the basis of what the patients reported during the phone calls/visits.

Pregnant and lactating women found positive at the screening test for CD were followed up in a different way, because they could not undergo all the diagnostic examinations needed to evaluate a possible cardiac/intestinal involvement (barium enema and chest X ray for instance) and could not take the treatment. Therefore, their data were included in this analysis only once they could complete the staging and receive the therapy (that is after they stopped to breastfeed).

### Statistical analysis

Data were analysed with Epi-Info program, version 7.1.0.6, Centers for Disease Control, August, 2012. Chi-squared test was used for dichotomous variables and t-test for continuous variables, as appropriate.

### Ethical issues

Written informed consent was required from the patients at the moment of the execution of the screening test. The study protocol was approved by the Ethics Committee (Comitato Etico della Provincia di Verona) on February 19^th^ 2013.

## Results

During the 9–year period, 332 patients were included in this study. Characteristics of the patients are described in table 1. The majority of the patients were women (73.9%) and the mean age was 41.8 years (range 11–71). Sixty-one per cent of the patients reported living in rural areas and 73.2% in mud houses, while 6.9% of them reported history of blood transfusion in endemic countries. Ninety-seven percent of the patients came from rural high-prevalence Bolivian environments, especially from Santa Cruz and Cochabamba Departments.

**Table 1:** Characteristics of the 332 patients with Chagas disease.

Age, years (mean)	41,8 (range 11–71)
Female sex	244 (73.9%)
**Country of origin:**	
Bolivia	323 (97.3%)
Argentina	3 (0.9%)
Ecuador	1 (0.3%)
Paraguay	1 (0.3%)
Brazil	2 (0.6%)
Venezuela	2 (0.6%)
Co-morbidities causing immunosuppression	5 (1.5%)
History of rural environments	205 (61.7%)
History of mud houses	243 (73.2%)
History of blood transfusion in endemic countries	23 (6.9%)
Time since arrival to Italy (years, mean)	6.9 (range 1–25)

Four patients were under immunosuppressant therapies: two for rheumatoid arthritis, one for psoriasis, one for systemic lupus erithematosus (SLE). One patient had acquired immunodeficiency syndrome (AIDS).

**Figure 1:**
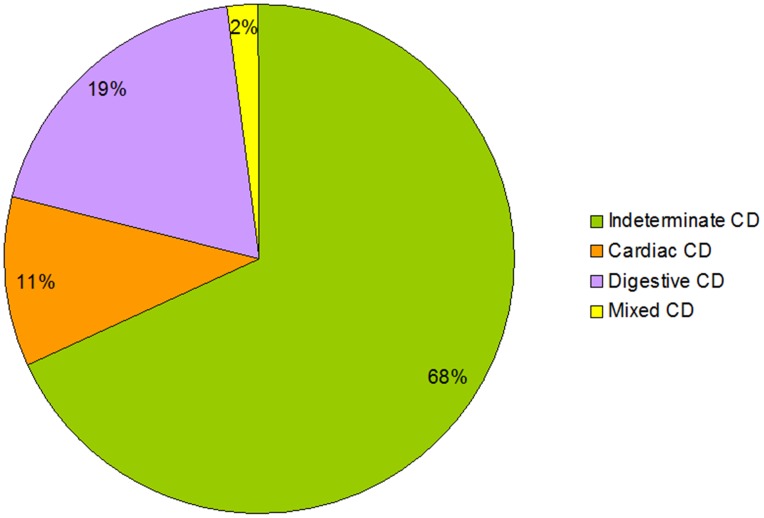
Classification of the 332 patients into different categories of Chagas disease.

According to the findings of instrumental exams, 226 (68.1%) subjects were classified as Indeterminate Chagas, 37 (11.1%) as Cardiac Chagas, 62 (18.7%) as Digestive Chagas, 7 (2.1%) as Mixed Form (Fig. 1).

Cardiac involvement is reported in table 2. In 288 patients (86.8%) ECG and echocardiogram resulted normal. Among patients with abnormal findings, the majority were classified in stage A (9%) or B1 (2%) (table 3 shows the ECG alterations found). The patient with AIDS showed abnormal ECG findings, abnormal echocardiogram findings with refractory CHF and needed cardiac transplantation. Four patients already had pacemaker at the time of diagnosis, while two needed pacemaker implantation at the time of the diagnosis or during the follow –up.

**Table 2:** Cardiac involvement in 332 patients with Chagas disease.

Findings	Stages	Patients	%
Normal ECG, normal echocardiogram	**0**	**288**	**86.8**
Abnormal ECG findings, normal echocardiogram findings, no signs of CHF	**A**	**30**	**9.0**
Abnormal ECG findings, abnormal echocardiogram findings with LVEF>45%, no signs of CHF	**B1**	**7**	**2.1**
Abnormal ECG findings, abnormal echocardiogram findings with LVEF <45%, no signs of CHF	**B2**	**2**	**0.6**
Abnormal ECG findings, abnormal echocardiogram findings, compensated CHF	**C**	**4**	**1.2**
Abnormal ECG findings, abnormal echocardiogram findings, refractory CHF	**D**	**1**	**0.3**

**Table 3:** Electrocardiographic alterations in 44 patients with Chagas disease.

Findings	Patients	%
Bradycardia (<50 bpm)	11	25.0
Right bundle branch block	10	22.7
Low voltages	6	13.7
First-degree atrioventricular block	5	11.4
Pacemaker	5	11.4
Tachyarrhythmia	3	6.8
Right bundle branch block and hemiblock	2	4.5
Q waves suggestive of necrosis or T waves changes suggesting ischemia	2	4.5

Gastrointestinal involvement is reported in table 4. At barium enema, dolichocolon was found in 33 patients (9.9%) and megacolon in 30 patients (9.0%). Oesophagogram resulted slightly pathological (group I) in 3 patients (0.9%). No patient was classified in group II and III. One patient was classified in group IV, because he underwent surgery for dolicho-megaoesophagus in Bolivia. One patient underwent multiple operations because of colonic volvulus. Two other patients presented mega-colon without toxic signs, but refused surgical operation and are followed with symptomatic treatment.

**Table 4:** Gastrointestinal involvement in 332 patients with Chagas disease.

Findings	Patients	%
**Barium enema**		
**normal**	269	81.1
**dolichocolon**: length from the anus to the transition to the descending colon>70 cm	33	9.9
**megacolon**: descending colon diameter>6,5 cm or ascending colon diameter>8 cm, or caecum diameter>12 cm	30	9.0
**Oesophagogram**		
**normal**	328	98.8
**group I**: oesophagus with normal calibre but with slow contrast progression and small retention of contrast 1 minute after ingestion	3	0.9
**group II**: oesophagus with small to moderate dilatation and considerable radiological contrast retention	0	0
**group III**: hypotonic oesophagus with important dilatation and great retention of contrast	0	0
**group IV**: oesophagus elongated that lies over the diaphragm with great retention of contrast (dolico-megaoesophagus)	1	0.3

Three hundred twenty-one patients (96.7%) were treated with benznidazole. Eleven patients (3.3%) were not treated because of refusal of treatment, or previous treatment in the country of origin.

Patients who presented at least one adverse effect were 89/321 (27.7%). In particular, 70 patients (21.8%) reported mild adverse effects, such as mild allergic skin reaction (54/70 patients), gastrointestinal adverse events (5/70), neurological or general adverse effects (headache, somnolence, weakness and arthromyalgia)(8/70), transaminase elevation (3/70); nineteen patients (5.9%) reported severe adverse effects, such as severe allergic skin reactions (7/19), peripheral neuropathy (6/19), leucopoenia (WBC <2500 cells/µL) (6/19). Patients who completed the treatment were 267/321 (83.2%). Among the 54 patients who had to stop the treatment, 19 had reported severe adverse effects, 26 mild allergic skin reactions, 3 gastrointestinal adverse effects, 5 neurological adverse effects, one had transaminase elevation. Two patients received nifurtimox as a second choice: one patient completed the therapy, the other one had to stop because of toxicity.

## Discussion

In our study we present 332 patients with CD who attended our Centre in a 9-year period. This represents the largest cohort of *T. cruzi* infected patients reported in Italy, taking into account that currently around 360 patients have been diagnosed and eventually treated in the whole of Italy (Non Endemic Country – “NEC” Initiative; meeting in Florence 2013).

Unfortunately, it has been estimated that around 9200 cases may be present in Italy [5], therefore an improvement of the access to diagnosis and cure is strongly needed. Lack of awareness both in the medical community and also in the majority of Latin American immigrants, scarceness of specialized facilities where screening tests are available, absence of specific National guidelines/recommendations are some of the main obstacles to reach this goal.

The majority of our patients (97.3%) came from Bolivia; this is due both to the fact that Bolivia is the country with the highest prevalence worldwide, and that our screening program was implemented with the Bolivian community in Northern Italy. The mean age was 41.8 years and 73.9% of the patients were women: these data reflect the profile of immigrants from South America, who are mostly young women working as caregivers for elderly Italians.

We had 4 patients under immunosuppressant therapies, but fortunately none of them presented reactivation of CD; on the other hand, the patient with AIDS had a reactivation of CD and needed heart transplantation. In this regard, the clinicians should consider the risk of reactivation of CD in immunosuppressed patients, with higher parasitaemia and life threatening manifestations, such as meningoencephalitis and myocarditis [17].

We found 13.2% of patients with cardiac involvement (cardiac and mixed form): this percentage is considerably lower than the data reported by the main case series of chronic CD patients in endemic areas [18]–[26] (mean 35.3%, range 20.3%–52.1%), but also lower than main case series in Europe (mean 24.5%, range 19.9%–49.0%) [16], [27]–[31]. The differences between endemic countries and Europe are probably due to the fact that in endemic areas symptomatic patients with cardiac involvement attend health centers more often than asymptomatic subjects, and that most Latin American immigrants are on average younger than the resident population. However, our rate of cardiac involvement is even lower than the ones reported by other European case series. The main reasons could be due to differences in the origin of the study populations, in the screening methods and classifications criteria. For instance, we decided not to consider clinical symptoms for the classification of heart disease but to rely only on instrumental parameters, because symptoms are nonspecific and hardly comparable [10]. Serological screening was offered to all the members of the Bolivian community in Bergamo province, therefore our data can be considered a more reliable picture of the entire population (about 22% of the screened population resulted positive for CD).

Also for gastrointestinal involvement we decided to classify the patients without considering the symptoms. In fact, most signs and symptoms of digestive involvement are non specific; moreover, patients with CD can present digestive disorders unrelated to this infection [13]. Our rate of patients with digestive involvement (around 20%) is similar to the ones reported by the main case series of chronic CD patients in endemic areas [18]–[26] (mean 20.4%, range 9.5%–35.1%), but significantly higher than main clinical series in Europe (mean 8.7%, range 0.7%–22%) [16], [27]–[31]. Dolichocolon and megacolon were the most frequent findings while oesophagus alterations were found in a very small percentage of patients. Our data are similar to the ones by Salvador *et al* [17] and therefore we reached the same conclusions: colonic radiological evaluation is useful for every patient, while systematic oesophageal assessment is probably not cost-effective in asymptomatic patients. Perez Ayala *et al* [32] suggested that oesophageal manometry may be more useful than barium swallow for the detection of early oesophageal involvement, as previously reported by Dantas [33]; he also postulated that early treatment with benznidazole may offer similar benefits in gastro-intestinal involvement as is reported in cardiomyopathy[34], but evidence is scarce [32].

The treatment for CD includes only two drugs, nifurtimox and benznidazole. The latter, in consideration of its better safety and efficacy profile, is used as first line treatment [15]. However benznidazole, too, can cause serious adverse events, moreover efficacy data are based only on a few studies [34], [35]. Although more evidence is needed, we considered potential toxicity less dangerous than the possible complications of the disease. We thus treated almost all subjects with a positive serologic result (96.7%): this percentage is higher if compared to other studies in non endemic countries. In our series 27.7% of the patients reported adverse effects (21.8% mild, and 5.9% severe). These rates are lower than those (40.2%) reported by Carrilero *et al* [36] and (52%) by Perez Ayala *et al* [16]. Patients who had to stop the treatment were 16.8% in our series, 29.7% in the case series by Perez Ayala *et al*, and 5.6% in that by Carrilero *et al*. However, data are very heterogeneous and need a standardisation to be correctly evaluated.

Some problems are still unsolved in Italy: the patients diagnosed and treated so far are only a small proportion of the estimated affected population and should be considered as a first step after “breaking the epidemiological silence” [6]. Considering that the majority of patients are asymptomatic, public health measures for the prevention of the transmission and improvement of the access to diagnosis and treatment should be implemented.

Until now no case of transmission of CD through blood transfusion or organ donation has been reported in Italy. One case of fatal reactivation after a bone marrow transplantation performed in Italy in an Argentinean girl was reported [37], as well as one case of mother-to-child transmission (Non Endemic Country – “NEC” Initiative; meeting in Florence 2013). Unfortunately this issue has not yet been officially addressed in our country, despite the profile of Latin American immigrants resident in Italy, that is worrisome in relation to the possibility of vertical transmission of CD. The only Italian Region that has implemented an official screening program targeted to Latin American pregnant women is Tuscany; in Bergamo area (Lumbardy), too, a screening program to control vertical transmission has been implemented since August, 2013. In the next months Veneto Region, too, will implement a pilot screening (Napoletano G, personal communication).

### Conclusions

CD is an example of a new challenge of global health and should be no longer perceived as an exotic disease [30], but one that is currently present in Italy. Public health policies (with the support of anthropologists) to reach migrants from Latin America are urgently needed in order to plan appropriate screening strategies to facilitate contacts with the communities of immigrants [30], test risk groups and offer the treatment to the patients with the infection.

Official European guidelines for CD management are still lacking (the Spanish ones are commonly followed [9], [13]). Moreover, different classifications (for cardiac and digestive involvement) are being used internationally. Efforts are necessary in order to uniform management strategies and classification criteria. Moreover, up to now, we do not have any evidence on the disease evolution in non endemic countries where vectorial re-infection is not possible and where there are different life styles and comorbidities.

## Supporting Information

S1 Checklist STROBE checklist.
(DOC)
